# The prognostic value of tumour stroma ratio and tumour budding in stage II colon cancer. A nationwide population-based study

**DOI:** 10.1007/s00384-018-3076-9

**Published:** 2018-05-21

**Authors:** Ann Christina Eriksen, Flemming B. Sørensen, Jan Lindebjerg, Henrik Hager, René dePont Christensen, Sanne Kjær-Frifeldt, Torben F. Hansen

**Affiliations:** 1 Danish Colorectal Cancer Center South, Vejle Hospital, Vejle, Denmark; 2 Institute of Regional Health Research, University of Southern Denmark, Odense, Denmark; 3 Department of Pathology, Lillebaelt Hospital, Beriderbakken 4, DK-7100 Vejle, Denmark; 4 Department of Clinical Medicine, University Institute of Pathology, Aarhus University Hospital, University of Aarhus, Aarhus, Denmark; 5 Danish Colorectal Cancer Group (DCCG), Hvidovre, Denmark; 6 Research Unit of General Practice, University of Southern Denmark, Odense, Denmark

**Keywords:** Colon cancer stage II, Prognostic markers, Survival, Tumour budding, Tumour stroma ratio

## Abstract

**Purpose:**

High-risk patients with stage II colon cancer (CC) may benefit from adjuvant chemotherapy, but additional prognostic markers are needed for better stratification. We investigated the prognostic value of tumour stroma ratio (TSR) and tumour budding (TB).

**Methods:**

A nationwide population-based cohort of 573 patients with stage II CC was included. TSR was scored on hematoxylin and eosin sections as low TSR (> 50% stroma) and high TSR (≤ 50% stroma). TB was evaluated in hotspots on pan-cytokeratin stained sections in 10 high power fields (HPF) at the invasive front and classified by the mean number of buds *per* HPF as high grade budding (≥ 10 buds) or low-grade budding (< 10 buds). The prognostic value was investigated in Cox proportional hazard models for recurrence-free survival (RFS) and overall survival (OS).

**Results:**

Low TSR was associated with worse RFS (HR = 1.342 (95% CI 1.006–1.791), *p* = 0.045) and OS (HR = 1.376 (95% CI 1.016–1.862), *p* = 0.039). Furthermore, an association was found between low TSR and microsatellite stabile tumours (*p* < 0.001). The mean number of buds *per* HPF was associated to TSR with increasing number of buds related to a lower TSR (*p* = 0.026). No statistically significant prognostic impact of TB regarding OS or RFS was detected.

**Conclusions:**

TSR provided valuable prognostic information, and adding TSR to the current risk stratification may contribute to better patient selection. The estimates of TSR and TB were found to be associated, but no prognostic value of TB was documented.

## Introduction

Colon cancer (CC) is one of the most prevalent cancers in the Western world, and approximately one third of the patients are diagnosed with stage II disease. This subgroup of patients has an overall good prognosis with a 5-year overall survival (OS) of approximately 70–80% after surgery alone [1], and the use of adjuvant chemotherapy is often questionable. Current international guidelines (ASCO and ESMO) recommend adjuvant treatment limited to patients at high risk of recurrence identified by at least one of the following clinical characteristics: pT4 tumours; extramural vascular, lymphatic or perineural invasion; poorly differentiated histology; and obstruction or tumour perforation and lymph node sampling lower than 12 [1, 2]. However, these risk factors have been found insufficient for ideal selection of patients for adjuvant therapy [3], and there is a need for additional prognostic markers for better clinical management of patients with stage II CC.

Stroma cells play a central role in the invasion-metastasis-cascade [4], the process where cancer cells detach from the primary tumour, invade the surrounding stroma, penetrate the blood vessel wall and travel with the bloodstream to form a metastasis at a distant location. Tumour stroma ratio (TSR) is an estimate of the proportion of epithelial and stromal cells, and several studies in colorectal cancer (CRC) have identified TSR to be an independent prognostic marker with a high amount of stroma (low TSR) being predictive of an adverse outcome [5–10]. However, the prognostic impact of TSR in a large unbiased population-based cohort of stage II CC is currently unknown.

Tumour budding (TB) is considered to be associated with epithelial mesenchymal transition (EMT) at the invasive front and thus might represent the cell-biological correlate of the tumour-stroma interphase. The potential relationship between TB and TSR, however, is sparsely mentioned in the literature. Numerous studies [11–17] and systematic reviews [18, 19] show prognostic information of TB in stage II CRC with a high number of tumour buds associated with adverse outcome, and The International Tumour Budding Consensus Conference Group recommends TB to be included in the high-risk stratification of stage II CRC [20]. However, few studies have investigated solely stage II CC [12, 15]. Furthermore, studies vary in methods. Often, TB is evaluated in hematoxylin and eosin (H&E) sections, and only few studies have investigated TB in pan-cytokeratin stained sections in stage II CC [17], although the inter-observer reproducibility is markedly improved compared to H&E [21].

The objectives of the present study were to evaluate the prognostic value of TSR and TB in a nationwide, population-based cohort of stage II CC and to investigate the relationship between TSR and TB.

## Methods

This study is reported in accordance with REMARK [22].

### Patient population

This retrospective study was based on a true national, population-based cohort. All patients with surgically resected stage II CC in Denmark in 2002 (*N* = 746) were identified by a search in the nationwide registry administered by the Danish Colorectal Cancer Group (DCCG). This database contains prospectively collected surgical and pathological data. All Danish departments of pathology agreed to participate in the study. Thus, the cohort represents the entire Danish population of stage II CC in 2002. The following exclusion criteria were applied: tumour blocks missing in archives (*N* = 11), insufficient tissue for analysis (*N* = 1), stage II not recognized in new sections cut (*N* = 21), suspicion of stage IV (*N* = 4), patients treated with adjuvant chemotherapy (*N* = 26)/radiotherapy (N = 1), and death within 90 days of surgery (*N* = 75). Patients diagnosed before primary CC with another malignancy except non-melanoma skin cancer were also excluded from the study (*N* = 26). Further, patients with loco-advanced disease (*N* = 8) were excluded, and the final study population comprised 573 patients.

Tumours located from the caecum to the transverse colon were defined as right-sided cancers, and tumours located from the left colonic flexure to the sigmoideum were defined as left-sided. Tumours from the rectum were not included.

Patients with histologically verified recurrence were identified through the national registry, PatoBanken, containing all pathology reports in Denmark. Patients diagnosed with other histologically verified malignancies were also identified using this registry and subsequently censored from the recurrence-free survival (RFS) analysis on the date of their new cancer diagnosis. Information on treatment with adjuvant chemotherapy, non-histologically verified recurrence, or diagnosis of other malignancies was obtained from the National Patient Registry containing information on all Danish citizens’ contacts to the health care system linked by the Danish social security number.

The follow-up period was 7 years from the date of surgery. This interval was selected, since most patients experienced recurrence within 5 years from being diagnosed with their primary disease. Information on T-category, malignancy grade, localization, number of lymph nodes removed, and neural and vascular invasion was obtained from the original pathology reports. In case of an inconclusive report on T-category or malignancy grade, the decision was made by microscopy of new sections. Information on tumour perforation, ileus, and type of surgery was obtained partly from the pathology reports and partly from the DCCG registry based on the surgeon’s report.

The study was approved by The Regional Committees on Health Research Ethics for Southern Denmark (S-20140119) and the Danish Data Protection Agency (14/26345). All patients were screened in the Danish Registry of Tissue Utilization before enrolment in the study.

### Samples

Archived formalin-fixed, paraffin-embedded tissue blocks were stored and transported at room temperature. One tumour block representing the deepest invasive front was selected from each patient by both a trainee and an experienced pathologist from the scientific group. Microsatellite instability (MSI) status was assessed by immunohistochemistry.

### Tumour stroma ratio

TSR was estimated on H&E stained sections in a semi-quantitative manner. Slides were scanned at ×2.5 or ×5 magnification, and the area with the highest fraction of tumour stroma was selected. Subsequently, TSR was assessed in this area at ×10 magnification in one field with tumour cells present at all borders (north-east-south-west) as described by Huijbers et al. [7]. The stroma percentage was estimated *per* microscopic field and scored into four groups: 1: TSR > 75%, 2: 50% < TSR ≤ 75, 3: 25% < TSR ≤ 50%, and 4: TSR ≤ 25%. The four-tiered data was categorized into two groups as high (> 50%) and low (≤ 50%) TSR as defined by Mesker et al. [5]. Whenever a score was difficult to settle in the selected area, the decision was guided by the overall impression of the stromal fraction in the tumour. Areas with necrosis were avoided. In mucinous tumours, the area with mucin was visually excluded from the scoring. Major vascular structures and muscle tissue were also visually excluded, whereas nerves, smaller vascular structures, and lymphocytic infiltration were not excluded from the stromal compartment.

### Tumour budding

The number of tumour buds was counted along the invasive front using pan-cytokeratin (AE1/AE3)-stained sections. First, the sections were examined at low magnification, and the area of the invasive margin representing the highest density of tumour buds was subjectively selected (hot-spot sampling). Afterwards, the number of tumour buds was counted in 10 HPFs at ×40 magnification. A bud was defined as an isolated single adenocarcinoma cell or a small cluster of up to four tumour cells as defined by Ueno et al. [23]. Adenocarcinoma cells were excluded from the counts, if they did not expose a clearly defined, blue, hematoxylin-stained nucleus, to avoid count of immunohistochemically stained, brown cytoplasmic fragments and artefacts. In sections with less than 10 HPFs available, buds were counted in as many adjacent HPFs as possible, and the mean number of buds was calculated according to this number of fields. Data were dichotomized using 10 buds *per* HPF as cut-off, and high-grade TB was defined as an average of ≥ 10 buds across 10 HPFs as recommended by Kamamitopoulou et al. [24].

### Immunohistochemistry

For immunohistochemical (IHC) analysis, 4 μm sections were mounted on FLEX IHC Microscope Slides (K8020, Agilent DAKO products, Glostrup, Denmark). The pretreatment processes were performed using PT Link (DAKO). Heat-induced epitope retrieval was achieved with Envision Target Retrieval Solution (DAKO) at pH 9 and 97 °C for 20 min, and staining was performed using a DAKO Autostainer Link 48 (DAKO). Endogenous peroxidase activity was blocked by Envision FLEX Peroxidase-Blocking Reagent (DAKO). The primary antibody was mouse monoclonal AE1/AE3 (code M3515, DAKO) diluted 1:250 with Envision Flex antibody diluent (code S2022, DAKO).

IHC staining of mismatch repair proteins was performed using monoclonal mouse antibodies: MLH1 (Novocastra, Leica, (Wetzlar) Germany, clone ES05, dilution 1:100, product code NCL-L-MLH1), MSH2 (Novocastra, Leica, clone 25D12, dilution 1:100, product code NCL-L-MSH2), MSH6 (BD Transduction Laboratories, clone 44/MSH6, dilution 1:200, material number 610919) and PMS2 (BD Pharmingen, clone A16-4, dilution 1:500, material number 556415).

The primary antibody was incubated for 30 min at room temperature, and for amplification, Envision Flex+ Mouse (Linker) (DAKO) was used for 20 min. Bound antibodies were detected by Envision FLEX/HRP (DAKO) and visualized by Envision FLEX DAB (DAKO) with chromogen diluted in Envision Flex Substrate Buffer (DAKO). Meyer’s hematoxylin (Merck, Darmstadt, Germany) was used as counterstain. To enhance the IHC stains of mismatch repair proteins, the sections were incubated in 0.5% CuSO_4_ in TBS buffer pH 7.6 for 10 min.

### Statistics

Overall survival (OS) was defined as the time between date of primary surgery and date of death from any cause or the date of last follow-up. Recurrence-free survival (RFS) was defined as the time from date of primary surgery until date of death of any cause or the date of first loco-regional or distant recurrence. Patients diagnosed with another cancer were censored at the date of that diagnosis. The Kaplan-Meier method was used to present survival curves, and the log-rank test was used to test for significant differences in survival time among the groups. We used the multivarable Cox-regression model with a hazard ratio (HR) of 1.0 as reference and a 95% confidence interval (CI). A cut-off significance level of 0.10 from a univariable Cox regression was prespecified for a parameter to be included in the multivariable Cox regression model.

Analyses of associations between clinicopathological variables were carried out by using *χ*^2^-tests. The statistical analyses were performed using the STATA software version 14.0 (StataCorp, Texas, USA), and all statistical tests were two-sided.

## Results

### Patient characteristics

Patient characteristics are summarized in Table 1. During the follow-up period of 7 years, 266 patients (46.4%) had died, 110 patients (19.2%) recurred and 78 patients (13.6%) had been diagnosed with another cancer. The median age at the time of surgery was 73 years (range 29–95), and the mean follow-up time was 6.9 years (range 3–84 months).

**Table 1:** Clinicopathological characteristics and association with tumour stroma ratio and tumour budding

	Number (*n* = 573) (%)	Tumour stroma ratio			Tumour budding		
		High	Low	*p* value	Low	High	*p* value
Age (years) at diagnosis							
Median	73						
Range	29–95						
≥ 73	268 (47)	181 (45)	87 (51)	0.144	251 (47)	17 (43)	0.575
< 73	305 (53)	223 (55)	82 (49)		282 (53)	23 (57)	
Gender							
Male	284 (50)	207 (51)	77 (46)	0.215	267 (50)	17 (43)	0.354
Female	289 (50)	197 (49)	92 (54)		266 (50)	23 (57)	
T-stage							
pT3	501 (87)	360 (89)	141 (83)	0.062	467 (88)	34 (85)	0.630
pT4	72 (13)	44 (11)	28 (17)		66 (12)	6 (15)	
Histology (WHO)							
Adenocarcinoma NOS	516 (90)	354 (87)	162 (96)	*0.010*	477 (89)	39 (97)	0.263
Mucinous adenocarcinoma	55 (10)	48 (12)	7 (4)		54 (10)	1 (3)	
Signet-ring cell carcinoma	2 (0.4)	2 (1)	0		2 (0.4)	0	
Malignancy grade							
Medium + low	451 (79)	309 (76)	142 (84)	*0.044*	428 (80)	23 (57)	*0.001*
High^a^	122 (21)	95 (24)	27 (16)		105 (20)	17 (43)	
Localization							
Right	273 (48)	200 (49)	73 (43)	0.168	249 (47)	24 (60)	0.105
Left	300 (52)	204 (51)	96 (57)		284 (53)	16 (40)	
Tumour perforation							
Yes	531 (93)	375 (98)	156 (95)	0.117	496 (97)	35 (92)	0.077
No	17 (3)	9 (2)	8 (5)		14 (3)	3 (8)	
Unknown	25 (4)						
Lymph nodes							
Median	10						
Range	0–41						
< 12 nodes	352	239 (59)	113 (67)	0.084	329 (62)	23 (58)	0.596
≥ 12 nodes	221	165 (41)	56 (33)		204 (38)	17 (42)	
Perineural invasion							
Yes	26 (5)	9 (3)	17 (14)	*< 0.001*	25 (7)	1 (4)	0.543
No	360 (63)	254 (97)	106 (86)		335 (93)	25 (96)	
Not assessed	187 (32)						
Vascular invasion							
Yes	43 (7)	23 (8)	20 (15)	*0.027*	39 (10)	4 (14)	0.481
No	387 (68)	271 (92)	116 (85)		362 (90)	25 (86)	
Not assessed	143 (25)						
Mismatch repair status							
MSS	400 (70)	264 (65)	136 (80)	*< 0.001*	377 (71)	23 (58)	0.079
MSI	173 (30)	140 (35)	33 (20)		156 (29)	17 (42)	

Significant results in italics

*MSI* microsatellite instability, *MSS* microsatellite stable, *NOS* not otherwise specified, *T* tumour

^a^Including mucinous adenocarcinomas and signet-ring cell carcinomas

### Tumour stroma ratio

TSR was scored as 1 in 230 (40.1%) cases, 2 in 174 (30.4%) cases, 3 in 151 (26.4%) cases and as 4 in 18 (3.1%) cases. After dichotomizing the data with a cut-off of 50%, a total of 404 (70.5%) tumours were categorized as high TSR and 169 (29.5%) as low TSR.

### Tumour budding

The mean and median number of buds *per* HPF were 4.5 ± 3.7 (SD) (range 0–25) and 3.5, respectively. In two cases, it was not possible to count TB in 10 HPFs. Using 10 buds *per* HPF as cut-off, 533 (93.0%) of the cases were classified as low-grade budding, whereas 40 (7.0%) were classified as high-grade budding. Using five buds *per* HPF as cut-off, the number of cases classified as high-grade budding was increased to 186 (32.5%).

### Correlation between the investigated parameters

**Fig. 1:**
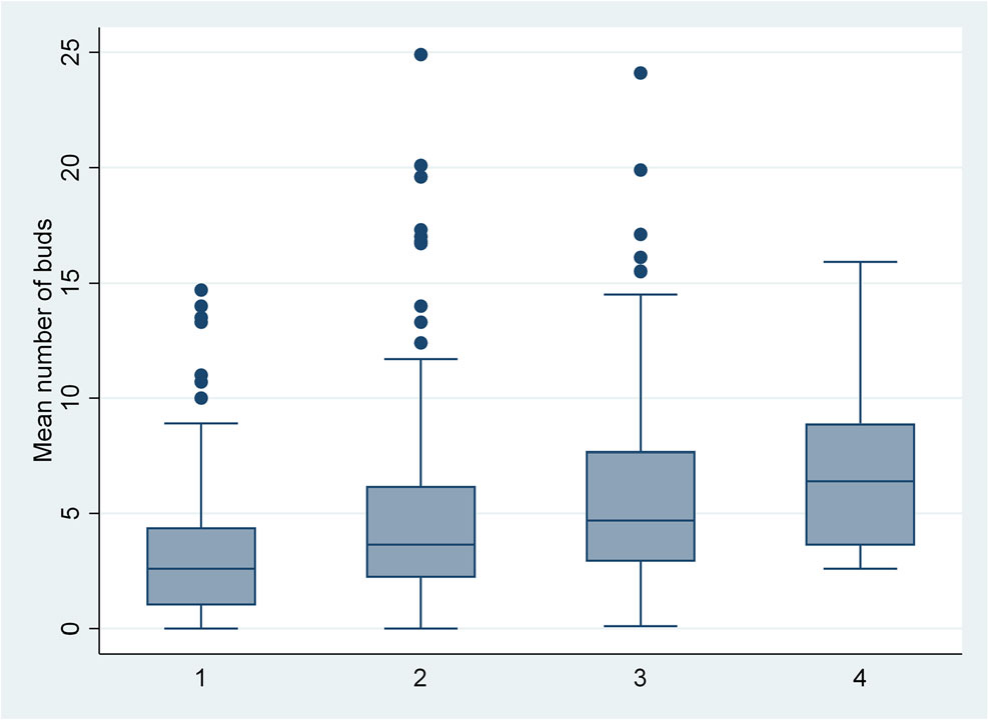
Correlation between tumour stroma ratio and tumour budding. Boxplot of mean number of buds *per* HPF in the four different groups of tumour stroma ratio (TSR); 1: TSR > 75%, 2: 50% < TSR ≤ 75, 3: 25% < TSR ≤ 50, and 4: TSR ≤ 25%. Box-plot charts (50% of values within the box; horizontal bar represents the median and vertical bar range of values), *p* = 0.013

We found a significant correlation between the mean number of buds *per* HPF and TSR with an increasing number of tumour buds related to a lower TSR (Fig. 1). The relationship between clinicopathological characteristics, TSR and TB is presented in Table 1. Low TSR was associated with the histological type of adenocarcinoma NOS (*p* = 0.010), medium/low malignancy grade, perineural invasion (*p* < 0.001), vascular invasion (*p* = 0.027) and MSI status (*p* < 0.001), while low-grade TB was only associated with medium/low malignancy grade (*p* = 0.001) and a near significant association with MSI (*p* = 0.079).

### Survival analyses

**Fig. 2:**
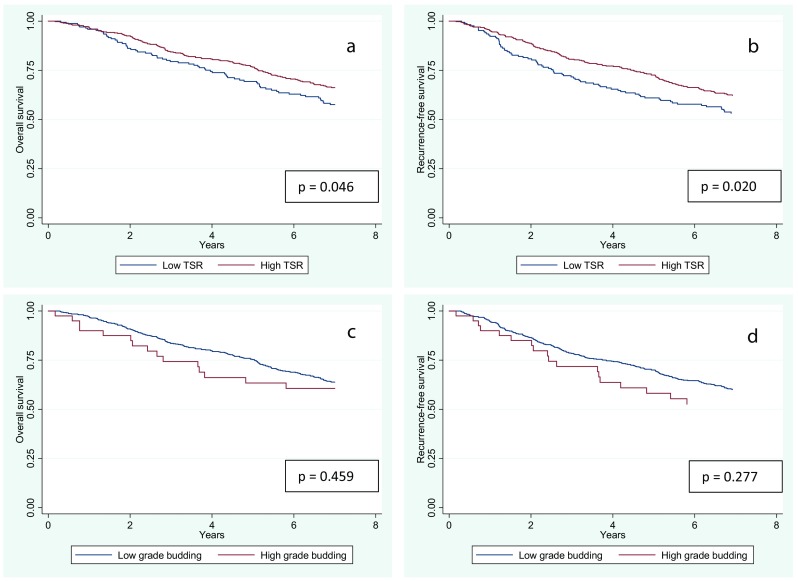
Kaplan-Meier survival curves for overall survival (OS) and recurrence-free survival (RFS). **a**, **b** High tumour stroma ratio (TSR) versus low TSR. **c**, **d** High-grade budding versus low-grade budding in the total patient population (*n* = 573). Log-rank test was used to test for significant survival time differences among groups

In the TSR-low population, the 5-year RFS rate was 61.0 versus 72.6% in the TSR-high population. OS was 69.4 and 76.7% in the TSR-low and high populations, respectively (Fig. 2a, b). Significant differences were observed for both RFS (*p* = 0.0204) and OS (*p* = 0.0460).

The 5-year RFS rate was 58.2% in the high-grade TB group versus 70.0% in the low-grade TB group, and the corresponding 5-year OS was 63.4 and 75.3%, respectively (Fig. 2c, d). No significant differences were observed for either RFS (*p* = 0.277) or OS (*p* = 0.459).

Results from the univariable and multivariable Cox regression analyses are shown in Tables 2 and 3. In the univariable analysis, T4 tumour and perforation were significantly related to poor OS, and moreover, localization and MSI were nearly significantly related to RFS. Low TSR was significantly related to an adverse outcome of both RFS and OS. No significant prognostic impact of TB regarding RFS or OS was found. Additional survival analysis was performed using five buds *per* HPF as cut-off; however, this did not provide any prognostic information (data not shown).

**Table 2:** Cox regression analysis, overall survival (*n* = 573)

Parameter	Simple analysis		Multiple analysis	
	HR (95% CI)	*p* value	HR (95% CI)	*p* value
Age				
< 73	1	*< 0.001*	1	*< 0.001*
≥ 73	2.405 (1.774–3.260)		2.757 (2.005–3.792)	
Sex				
Male	1	0.448	–	–
Female	0.897 (0.678–1.187)			
T-category				
T3	1	*0.007*	1	*0.003*
T4	1.662 (1.148–2.407)		1.785 (1.218–2.615)	
Malignancy grade				
Medium/low	1	0.292	–	–
High^a^	1.193 (0.859–1.656)			
Localization				
Right	1	0.201	–	–
Left	1.202 (0.907–1.593)			
Tumour perforation				
No	1	*0.033*	1	*0.011*
Yes	2.072 (1.059–4.052)		2.440 (1.227–4.851)	
Lymph nodes				
< 12 nodes	1	0.295	–	–
≥ 12 nodes	0.856 (0.640–1.145)			
Perineural invasion				
No	1	0.153	–	–
Yes	1.540 (0.851–2.786)			
Vascular invasion				
No	1	0.409	–	–
Yes	1.237 (0.747–2.047)			
Mismatch repair status				
MSS	1	0.272	–	–
MSI	0.839 (0.613–1.148)			
TSR				
High	1	*0.047*	1	*0.039*
Low	1.348 (1.004–1.809)		1.376 (1.016–1.862)	
Budding				
Low	1	0.460	–	–
High	1.220 (0.720–2.065)			

Significant results in italics

*MSI* microsatellite instability, *MSS* microsatellite stable, *T* tumour

^a^Including mucinous adenocarcinomas and signet-ring cell carcinomas

**Table 3:** Cox regression analysis, recurrence-free survival (*n* = 573)

Parameter	Simple analysis		Multiple analysis	
	HR (95% CI)	*p* value	HR (95% CI)	*p* value
Age				
< 73	1	*< 0.001*	1	*< 0.001*
≥ 73	1.854 (1.405–2.446)		2.096 (1.570–2.799)	
Sex				
Male	1	0.440	–	–
Female	0.901 (0.692–1.174)			
T-category				
T3	1	*0.009*	1	*0.010*
T4	1.612 (1.128–2.305)		1.622 (1.122–2.344)	
Malignancy grade				
Medium/low	1	0.820	–	–
High^a^	1.038 (0.754–1.429)			
Localization				
Right	1	0.094	1	0.156
Left	1.256 (0.962–1.639)		1.125 (0.917–1.713)	
Tumour perforation				
No	1	*0.010*	1	*0.008*
Yes	2.301 (1.218–4.349)		2.393 (1.253–4.571)	
Lymph nodes				
< 12 nodes	1	0.453	–	–
≥ 12 nodes	0.901 (0.685–1.184)			
Perineural invasion				
No	1	0.112	–	–
Yes	1.585 (0.898–2.799)			
Vascular invasion				
No	1	0.708	–	–
Yes	1.100 (0.667–1.816)			
Mismatch repair status				
MSS	1	0.057	1	0.304
MSI	0.746 (0.552–1.009)		0.828 (0.577–1.187)	
TSR				
High	1	*0.021*	1	*0.045*
Low	1.387 (1.051–1.832)		1.342 (1.006–1.791)	
Budding				
Low	1	0.278	–	–
High	1.306 (0.806–2.115)			

Significant results in italics

*MSI* microsatellite instability, *MSS* microsatellite stable, *T* tumour

^a^Including mucinous adenocarcinomas and signet-ring cell carcinomas

In the multivariable analysis, low TSR was associated with reduced RFS, HR = 1.342 (95% CI 1.006–1.791, *p* = 0.045), independent of age, T-stage, localization, perforation and MSI status. There was an independent significant impact of TSR on OS, HR = 1.376 (95% CI 1.016–1.862, *p* = 0.039). Age, T-stage and perforation were the only other parameters significantly related to OS and RFS in the multivariable analysis.

## Discussion

In the present study, we investigated the prognostic impact of TSR and TB in an unbiased, nationwide, population-based cohort of stage II CC patients treated exclusively with surgery.

We found an independent prognostic value of TSR according to both RFS and OS, which is in accordance with previous studies. Several studies have reported an intratumoural stroma-epithelium ratio or tumour stromal percentage to be an independent prognostic marker in CRC. However, conclusions on stage II CC are not easily extracted, as these studies included several disease stages [5, 7, 25], and some also included rectal cancer [6, 8, 9]. Recently, Hutchins et al. [9] quantified the relative proportion of stroma morphometrically in the QUSAR study cohort of stage II and III CRC patients, and they found tumours of the rectum to have a significantly higher stroma-ratio compared to those of the colon. Moreover, lower scores of stroma were found in stage II than in stage III. Thus, the impact of TSR in stage II CC should be considered separately. Hutchins et al. [9] conducted additional analyses of stage II CRC only and reported an adverse prognostic impact of a high stroma fraction. Also, Huijbers et al. reported a prognostic value for a subgroup of stage II CC; however, they found a slightly greater impact on both OS and DFS [7]. We investigated a true, population-based cohort exclusively consisting of patients resected for stage II CC, and to avoid possible confounding we excluded patients treated with adjuvant chemotherapy and patients with other known malignancies. Also, eight patients with loco-advanced disease were excluded. Consequently, our group of patients is highly homogeneous in contrast to other studies.

We did not find any significant association between TB and OS or RFS, which is in agreement with a study restricted to early stage CC (I, IIA, and IIB) by Gilardoni et al. [26] but contradictory to other studies of stage II CRC that have found high TB to be associated with decreased OS [12, 14] and disease-free survival (DFS) [17]. However, the published studies are highly variable in both study populations and TB assessment methods. Most studies evaluate TB on H&E sections and in one single HPF. Recently, Koelzer et al. [17] evaluated TB in a cohort of 150 stage II CRC patients using AE1/AE3 stained sections and the 10 HPFs method. They found high TB (≥ 9) to be an independent predictor of worse DFS, which is in contrast to our results. A possible explanation might be differences in study cohorts and methods. We investigated a population-based patient cohort of exclusively stage II CC, while Koelzer et al. also included upper rectal tumours. Furthermore, they selected the tissue section with the highest number of buds presented on H&E for IHC staining and evaluation of TB, whereas we used the section representing the deepest invasive margin. We have previously found heterogeneity of TB to be modest in stage II CC [27], and thus the use of different sections may only partly explain the disagreement in the obtained results.

Reported proportions of high-grade budding in stage II tumours vary from 19.5 [11] to 45% [13]. This may reflect both the heterogeneity of stage II tumours and differences in the evaluation methods and cut-offs applied. In our cohort, we only found 7.0% of the tumours to have high-grade TB. Patients having received adjuvant chemotherapy were excluded from our analysis, and since these patients have tumours expected to harbour a more aggressive biology, this may be part of the explanation. We counted tumour buds in 10 HPFs, and counting in multiple fields may ‘dilute’ the final mean number of buds, especially in tumours with focally high-grade tumour buds. Koelzer et al. [17] also counted in multiple fields and used the same cut-off; however, they found 30.7% of the tumours to have high TB. In order to enhance our proportion of high-grade budding tumours, we did an additional analysis using a lower cut-off (e.g. five buds/HPF). This yielded a higher proportion of tumours with high-grade budding, but it did not change the results of the survival analysis (data not shown), and for comparability with other studies, we chose to use the cut-off value of 10 buds *per* HPF. Koelzer et al. reported a mean of 7.11 buds *per* HPF. Our mean was lower, 4.5 ± 3.7 buds *per* HPF. We only counted TB cells with a clear, blue nuclear stain to avoid cytoplasmic fragments etc., which may be the reason for our lower TB count. We reported a kappa value of 0.73 for inter-observer agreement [27], while Koelzer et al. reported an ICC of 0.79 for four observers. Thus, the difference may be between centres and future studies using IHC should standardize the counting technique.

Koelzer et al. [17] did not find T-category to be independently associated to DFS, but we found this measure to be an independent predictor of both RFS and OS. This also reflects the challenges in stage II CC and comparing cohorts.

We found low TSR significantly associated to patients with microsatellite stable (MSS) tumours, a group of patients known to have a worse prognosis compared to patients with MSI tumours [28]. This might be related to the high occurrence of stroma and is in accordance with the Consensus Molecular Subtypes (CMS) [29], CMS4 representing the mesenchymal group. In this group, up to 92% of the tumours have been found to be MSS, and clinically, they are reported to have a worse RFS and OS [30]. The majority of CCs are MSS, and in future studies, special focus should be paid to the stroma.

We found a correlation between low TSR and high TB, which is consistent with a study reporting high-level TB to be significantly associated with increased tumour stroma [25]. This is in accordance with the consideration of both markers to reflect EMT. Recently, the relationship between TB and EMT has been questioned [31], and TB has been considered as a partial EMT [32]. This raises the question of how TB and tumour stroma might be related. Potentially, TSR and TB reflect the same changes in the microenvironment and do not add any prognostic information to one another. In that case, the question is which parameter is more applicable for clinical use. In our study, we could not demonstrate any significant prognostic value of TB, and thus TSR seems to be a better marker in the clinical setting. We found similar reproducibility for TSR (kappa = 0.75) and TB (kappa = 0.73), when counting TB on pan-cytokeratin stained sections and using the mean of tumour buds counted in 10 HPFs [27]. However this method is time consuming and this outlines the advantages of using TSR, which is easily estimated. Nevertheless additional research should be undertaken to further investigate the potential relationship between TSR and TB.

The present study is limited by the retrospective design. Data on recurrence and death have been obtained from registers, and the individual patient records have not been evaluated. Especially, the cause of death may be questionable, and for this reason, we did not use a cancer-specific endpoint. Patients were treated according to current guidelines back in 2002, which cause differences compared to a similar cohort of stage II CC treated today. However, the cohort represents the entire population of stage II CC treated in Denmark back in 2002 as thus represent a highly unbiased population.

## Conclusion

We found TSR to be of prognostic importance in a nationwide, population-based group of patients with stage II CC. Also, inclusion of TSR in the current risk-score for stage II CC may provide better stratification for adjuvant treatment. An association between TSR and TB was found with an increasing number of tumour buds related to a higher fraction of stroma. We did not find any significant prognostic value of TB.

## References

[1] Labianca R, Nordlinger B, Beretta GD (2013). Early colon cancer: ESMO clinical practice guidelines for diagnosis, treatment and follow-up. Ann Oncol.

[2] Benson AB, Schrag D, Somerfield MR (2004). American Society of Clinical Oncology recommendations on adjuvant chemotherapy for stage II colon cancer. J Clin Oncol.

[3] O'Connor ES, Greenblatt DY, LoConte NK (2011). Adjuvant chemotherapy for stage II colon cancer with poor prognostic features. J Clin Oncol.

[4] Pietras K, Ostman A (2010). Hallmarks of cancer: interactions with the tumor stroma. Exp Cell Res.

[5] Mesker WE, Junggeburt JM, Szuhai K, de Heer P, Morreau H, Tanke HJ, Tollenaar RA (2007). The carcinoma-stromal ratio of colon carcinoma is an independent factor for survival compared to lymph node status and tumor stage. Cell Oncol.

[6] West NP, Dattani M, McShane P, Hutchins G, Grabsch J, Mueller W, Treanor D, Quirke P, Grabsch H (2010). The proportion of tumour cells is an independent predictor for survival in colorectal cancer patients. Br J Cancer.

[7] Huijbers A, Tollenaar RA, v Pelt GW (2013). The proportion of tumor-stroma as a strong prognosticator for stage II and III colon cancer patients: validation in the VICTOR trial. Ann Oncol.

[8] Park JH, Richards CH, McMillan DC (2014). The relationship between tumour stroma percentage, the tumour microenvironment and survival in patients with primary operable colorectal cancer. Ann Oncol.

[9] Hutchins GGA, Treanor D, Wright A (2017). Intra-tumoural stromal morphometry predicts disease recurrence but not response to 5-fluorouracil—results from the QUASAR trial of colorectal cancer. Histopathology.

[10] Hynes SO, Coleman HG, Kelly PJ, Irwin S, O'Neill RF, Gray RT, McGready C, Dunne PD, McQuaid S, James JA, Salto-Tellez M, Loughrey MB (2017). Back to the future: routine morphological assessment of the tumour microenvironment is prognostic in stage II/III colon cancer in a large population-based study. Histopathology.

[11] Tanaka M, Hashiguchi Y, Ueno H, Hase K, Mochizuki H (2003) Tumor budding at the invasive margin can predict patients at high risk of recurrence after curative surgery for stage II, T3 colon cancer. Dis *Colon rectum* 46:1054–1059 10.1007/s10350-004-7280-z 12907899

[12] Nakamura T, Mitomi H, Kanazawa H, Ohkura Y, Watanabe M (2008) Tumor budding as an index to identify high-risk patients with stage II colon cancer. Dis *Colon rectum* 51:568–572 10.1007/s10350-008-9192-9 18286339

[13] Wang LM, Kevans D, Mulcahy H, OʼSullivan J, Fennelly D, Hyland J, OʼDonoghue D, Sheahan K (2009). Tumor budding is a strong and reproducible prognostic marker in T3N0 colorectal cancer. Am J Surg Pathol.

[14] Betge J, Kornprat P, Pollheimer MJ, Lindtner RA, Schlemmer A, Rehak P, Vieth M, Langner C (2012). Tumor budding is an independent predictor of outcome in AJCC/UICC stage II colorectal cancer. Ann Surg Oncol.

[15] Lai YH, Wu LC, Li PS, Wu WH, Yang SB, Xia P, He XX, Xiao LB (2014). Tumour budding is a reproducible index for risk stratification of patients with stage II colon cancer. Color Dis.

[16] Barresi V, Reggiani Bonetti L, Ieni A, Branca G, Tuccari G (2016). Histologic prognostic markers in stage IIA colorectal cancer: a comparative study. Scand J Gastroenterol.

[17] Koelzer VH, Assarzadegan N, Dawson H, Mitrovic B, Grin A, Messenger DE, Kirsch R, Riddell RH, Lugli A, Zlobec I (2017). Cytokeratin-based assessment of tumour budding in colorectal cancer: analysis in stage II patients and prospective diagnostic experience. J Pathol Clin Res.

[18] Rogers AC, Winter DC, Heeney A, Gibbons D, Lugli A, Puppa G, Sheahan K (2016). Systematic review and meta-analysis of the impact of tumour budding in colorectal cancer. Br J Cancer.

[19] Petrelli F, Pezzica E, Cabiddu M, Coinu A, Borgonovo K, Ghilardi M, Lonati V, Corti D, Barni S (2015). Tumour budding and survival in stage II colorectal Cancer: a systematic review and pooled analysis. J Gastrointest Cancer.

[20] Lugli A, Kirsch R, Ajioka Y, Bosman F, Cathomas G, Dawson H, el Zimaity H, Fléjou JF, Hansen TP, Hartmann A, Kakar S, Langner C, Nagtegaal I, Puppa G, Riddell R, Ristimäki A, Sheahan K, Smyrk T, Sugihara K, Terris B, Ueno H, Vieth M, Zlobec I, Quirke P (2017). Recommendations for reporting tumor budding in colorectal cancer based on the international tumor budding consensus conference (ITBCC) 2016. Mod Pathol.

[21] Koelzer VH, Zlobec I, Berger MD, Cathomas G, Dawson H, Dirschmid K, Hädrich M, Inderbitzin D, Offner F, Puppa G, Seelentag W, Schnüriger B, Tornillo L, Lugli A (2015). Tumor budding in colorectal cancer revisited: results of a multicenter interobserver study. Virchows Arch.

[22] McShane LM, Altman DG, Sauerbrei W (2005). REporting recommendations for tumour MARKer prognostic studies (REMARK). Br J Cancer.

[23] Ueno H, Murphy J, Jass JR, Mochizuki H, Talbot IC (2002). Tumour ‘budding’ as an index to estimate the potential of aggressiveness in rectal cancer. Histopathology.

[24] Karamitopoulou E, Zlobec I, Koelzer V (2013). Proposal for a 10-high-power-fields scoring method for the assessment of tumor budding in colorectal cancer. Mod Pathol.

[25] van Wyk HC, Park JH, Edwards J, Horgan PG, McMillan D, Going JJ (2016). The relationship between tumour budding, the tumour microenvironment and survival in patients with primary operable colorectal cancer. Br J Cancer.

[26] Gilardoni E, Bernasconi DP, Poli S, Garancini M, Luperto M, Zucchini N, Bovo G, Totis M, Bugatti A, Gianotti L (2015). Surveillance for early stages of colon cancer: potentials for optimizing follow-up protocols. World J Surg Oncol.

[27] Eriksen AC, Andersen JB, Lindebjerg J, dePont Christensen R, Hansen TF, Kjær-Frifeldt S, Sørensen FB (2018). Does heterogeneity matter in the estimation of tumour budding and tumour stroma ratio in colon cancer?. Diagn Pathol.

[28] Merok MA, Ahlquist T, Royrvik EC (2013). Microsatellite instability has a positive prognostic impact on stage II colorectal cancer after complete resection: results from a large, consecutive Norwegian series. Ann Oncol.

[29] Guinney J, Dienstmann R, Wang X, de Reyniès A, Schlicker A, Soneson C, Marisa L, Roepman P, Nyamundanda G, Angelino P, Bot BM, Morris JS, Simon IM, Gerster S, Fessler E, de Sousa E Melo F, Missiaglia E, Ramay H, Barras D, Homicsko K, Maru D, Manyam GC, Broom B, Boige V, Perez-Villamil B, Laderas T, Salazar R, Gray JW, Hanahan D, Tabernero J, Bernards R, Friend SH, Laurent-Puig P, Medema JP, Sadanandam A, Wessels L, Delorenzi M, Kopetz S, Vermeulen L, Tejpar S (2015). The consensus molecular subtypes of colorectal cancer. Nat Med.

[30] Thanki K, Nicholls ME, Gajjar A, Senagore AJ, Qiu S, Szabo C, Hellmich MR, Chao C (2017). Consensus molecular subtypes of colorectal cancer and their clinical implications. Int Biol Biomed J.

[31] Yamada N, Sugai T, Eizuka M, Tsuchida K, Sugimoto R, Mue Y, Suzuki M, Osakabe M, Uesugi N, Ishida K, Otsuka K, Matsumoto T (2017). Tumor budding at the invasive front of colorectal cancer may not be associated with the epithelial-mesenchymal transition. Hum Pathol.

[32] Grigore AD, Jolly MK, Jia D, Farach-Carson M, Levine H (2016). Tumor budding: the name is EMT. Partial EMT. J Clin Med.

